# Body fat percentage in adolescents with phenylketonuria and associated factors

**DOI:** 10.1016/j.ymgmr.2020.100595

**Published:** 2020-05-12

**Authors:** Giovanna Caliman Camatta, Viviane de Cássia Kanufre, Michelle Rosa Andrade Alves, Rosângelis Del Lama Soares, Rocksane de Carvalho Norton, Marcos José Burle de Aguiar, Ana Lúcia Pimenta Starling

**Affiliations:** a Associated research in Núcleo de Ações e Pesquisa em Apoio Diagnóstico (NUPAD), School of Medicine, Federal University of Minas Gerais (UFMG), Av. Prof. Alfredo Balena, 189, 30.130-100 Belo Horizonte, Brazil; b Postgraduate Program in Pediatrics and Adolescent Health, School of Medicine, UFMG, Av. Alfredo Balena 110, Santa Efigênia, 30.130-100 Belo Horizonte, Brazil; c Hospital das Clínicas, UFMG, Av. Alfredo Balena 110, Santa Efigênia, 30.130-100 Belo Horizonte, Brazil; d School of Medicine, UFMG, Av. Alfredo Balena 110, Santa Efigênia, 30.130-100 Belo Horizonte, Brazil

**Keywords:** Phenylketonuria, Adolescent, Body composition, Physical activity, Diet

## Abstract

**Objective:**

To evaluate the percentage of body fat (% BF) in adolescents with PKU and to relate it to protein consumption, physical activity level, body mass index (BMI), sexual maturity and metabolic control.

**Method:**

This is a cross-sectional study conducted with 94 adolescents between 10 and 20 years of age, with early diagnosis and continuous treatment. Bioimpedance, weight measurements, height and BMI calculation were performed. Questionnaires were applied to quantify protein ingestion and establish the level of physical activity. Sexual maturity was assessed using the Tanner criteria. The annual mean of serum phenylalanine was used as a control parameter of the disease. A multivariate linear regression analysis was performed.

**Results:**

Overweight, obesity, the female sex and the percentage of protein consumption explain 94.1% of the % BF of the patients (*p* < .05). The overweight prevalence was 19.1%. It was verified that 96.7% of the sample were sedentary. Only 50 (53.2%) of the adolescents had good treatment compliance, and no relationship was found between this variable and the % BF (*p* = .706).

**Conclusions:**

Being female and presenting high BMI values are important factors associated with % BF in phenylketonuric adolescents. Disease control and protein consumption do not seem to influence the body composition.

## 1 Introduction

Phenylketonuria (PKU) is an inborn error of metabolism caused by the deficiency or insufficiency of the enzyme phenylalanine hydroxylase (PAH), responsible for converting the amino acid phenylalanine (phe) into tyrosine (tyr) [1,2]. The increase in the blood concentration of phe can lead to irreversible damage to the nervous system and the impaired development of affected individuals, the most dramatic outcome being mental retardation [3,4].

Treatment is based on a strict low-phe diet supported by the use of a protein substitute – usually an amino acid mix – containing no phe or a small amount of it [1,5]. Such diet is for life and should be introduced as early as the 10th day of life or sooner [6]. As a result of this low-phe diet, the ingestion of natural protein is very restricted and the protein substitute accounts for 85% to 90% of total protein intake, and may also supply some vitamins and minerals [1].

Studies have suggested that a restrictive diet combined with high intake of sugars and the severity of PKU (depending on the blood concentrations of phe in the absence of treatment) can lead to weight gain in those individuals [7,8]. At puberty, a period of intense organic changes and anabolic effects, a high-protein diet may lead to lean mass gain, while a high-calorie diet may cause fat gain and poor nutritional status [[9], [10], [11]]. Thus, patients with PKU, especially in adolescence, may be more exposed to body composition deviations because of their diets [7,8,12].

Some studies have found that, among individuals with PKU, those with poorer metabolic control are more likely to be overweight than others, but such findings have not been supported elsewhere [7,8,13]. Moreover, a relevant factor in determining body composition is the practice of physical activity. Although this has been widely investigated in the general population, studies have neglected to consider the level of physical activity in adolescents with PKU. Only one study has tried to fill this gap and has found low levels of activity in this population [14].

Considering the lack of consensus relating PKU diet and nutritional status, the present study aims to investigate the body composition of adolescents with PKU and potential associated factors, including metabolic control of the disease, protein intake, physical activity level, sex and stage of sexual maturity. The study was done with subjects from a reference center in Brazil.

## 2 Methodology

A cross-sectional, observational study was carried out from August 2015 to July 2016 and comprised a sample of 10- to 20-year-old adolescents diagnosed with PKU within their first 30 days of life [15]. Exclusion criteria included: tetrahydrobiopterin (BH_4_) deficiency, use of pacemaker, pregnancy, growth-related disorder, and abandonment of treatment over the two previous years, which means being absent from clinical follow-up for more than one year, regardless of active search done by social workers. All participants were under treatment at the Phenylketonuria Outpatient Clinic of the Special Service of Genetics at the UFMG University Hospital. The study was approved by the university's ethics committee, and informed consent was obtained from all legal adults and legal guardians of minors.

All adolescents in the program have their natural protein and protein substitute consumption adjusted by dietitians every six months, during periodic follow-up appointments. Medical food is composed by a mix of phenylalanine-free l-amino acids enriched with micronutrients. The recommended amount of protein substitute varies according to individual tolerance and includes fractioning into 3 to 5 doses a day. Service protocol states the consumption of 1.0 to 1.5 g/kg/day of total protein per individual >10 years old, based on DRI recommendations [16] increased by 50%. No dietetic prescription is made for energy, carbohydrate or lipid amounts, except for those individuals with overweight/obesity and/or metabolic disorder. Special low-protein foods are not provided by the program and are usually purchased or prepared by families.

The outcome variable was the percentage of body fat. The measurements were performed by using the tetrapolar bioelectrical impedance system Biodynamics® 450. Anthropometric measurements were based on weight and height values collected on site by a single examiner. All participants were previously instructed to follow the protocol proposed by Biodynamics®, which recommends not doing intense physical activity, not consuming caffeine and not taking diuretic medication in the previous 24 h, as well as not consuming heavy meals 4 h before the data collection. Women should not be on their menstrual period. Data collection was postponed for participants who failed to adhere to the protocol.

Anthropometric indexes were classified using the WHO AnthroPlus® software (version 1.0.4). Individuals up to 19 years old had their BMI-for-age z-scores determined, while individuals aged 19 years and one day or more had their BMIs evaluated based on their absolute values. BMI was calculated by the formula: BMI = weight (kg) / height^2^ (m). The results were classified according to WHO guidelines [17]. Height-for-age z-score was determined according to a protocol by the Brazilian Ministry of Health [18].

Physical activity level was assessed using the Physical Activity Questionnaire for Older Children (PAQ-C), validated for the Brazilian population [19]. The questionnaire is a 7-day recall instrument that contains 9 questions about sports training and physical activity practice in school and in leisure time, including weekends. Hence, it was applied only during the school year and for those attending school. Questions were scored from 1 to 5, and the final score is the mean of the 9 questions. Adolescents with a final score lower than 3 were ranked as sedentary, while those with a score greater than or equal to 3 were ranked as active. The stage of sexual maturity was determined by the pediatric team based on the Tanner scale.

Metabolic control was assessed based on the arithmetic mean of the phe concentrations over the 12 preceding months. Individuals with a mean blood phe concentration ranging from 120 to 700 μmol/L were classified as adequate, while mean blood phe concentrations outside of this range were classified as inadequate [15,20,21].

Participants were previously instructed to complete a Food Record (FR) within the 72 h prior to data collection. An adapted semi-quantitative Food Frequency Questionnaire (FFQ) was also applied for data collection to assess food intake. The FFQ was developed by the Special Service of Genetics and designed to reflect the last 6 months of food consumption of individuals with PKU. The questionnaire comprised 57 food items and their portion sizes, as well as space to report additional information about medical food and special low-protein food consumption.

The actual amount of protein intake (from natural sources and from the amino acid mix) and total calories were quantified using software Dietpro version 5.0. Nutrient amounts were based on the Brazilian Table of Nutritional Composition [22] complemented by Sônia Tucunduva's Table [23].

Statistical analysis was performed using SPSS version 19.0. Descriptive statistical analysis included frequencies, means, standard deviations and medians. A univariate analysis was first conducted using *p* < .2. This step included correlation tests, one-way ANOVA, the Mann-Whitney *U* test, the T-Student test and the Jonckheere- Terpstra test, depending on the variable characteristic. All dependent variables related to the outcome variable were then included in the multivariate analysis at a significance level of 0.05.

## 3 Results

The Special Service of Genetics had 122 individuals in follow-up care who were potentially eligible for the study. Of them, 94 (77%) consented to participate and complied with the research protocol. The 28 (23%) remaining individuals were excluded because they did not provide consent (15), could not be located (12), or did not complete the research protocol (1). Mean age was 14 years for both male and female. Table 1 shows the descriptive results of the sample.

**Table 1:** Description of the sample of adolescents with PKU.

Categorical variables (*n* = 94)	Frequency	%
Sex		
Male	53	56.40
Female	41	43.60
Height for age⁎		
Low	3	3.37
Normal	86	96.63
BMI classification		
Underweight	4	4.26
Normal weight	72	76.60
Overweight	10	10.63
Obesity	8	8.51
Pubertal stage⁎⁎		
Prepubertal	6	6.52
Pubertal	86	93.48
Physical activity level⁎⁎⁎		
Active	3	3.30
Sedentary	89	96.70
Blood phe concentration		
Adequate	50	53.20
Inadequate	44	46.80

Physical activity level “active” included: “moderately active,” “active,” and “very active.” BMI: body mass index. Data reported as frequency and percentage (%).

⁎ *n* = 89.

⁎⁎ Two participants refused to be evaluated, *n* = 92.

⁎⁎⁎ Two participants did not attend school, *n* = 92.

Table 2 shows the measures of central tendency (means or medians) for the variables body fat percentage, weight, BMI, PAQ-C score, phe concentration, percentage of total protein intake relative to energy intake (TEI) and total protein intake in g/kg/day considering FFQ and FR.

**Table 2:** Description of the group of adolescents with PKU.

Continuous variables (*n* = 94)	MCT (MD)
Body fat percentage	18.25 ± 8.8
BMI (kg / m^2^)	20.05 (17.50–22.20)
Weight (kg)	52.15 (41.90–60.07)
PAQ-C score⁎	1.92 (1.63–2.23)
Mean blood phe (μmol / L)	661.70 ± 193.1
Total protein intake (% TEI)	
FFQ	12.61 (9.76–16.29)
FR	16.98 (13.09–21.60)
Mean	14.91 (11.90–18.16)
Total protein intake (g/kg/day)	
FFQ	1.44 (1.15–1.90)
FR	1.39 (1.05–1.70)
Mean	1.39 (1.09–1.77)

MCT (MD): measure of central tendency (measure of dispersion). BMI: body mass index. TEI: total energy intake. FFQ: Food Frequency Questionnaire. FR: Food Record. Data reported as mean ± standard deviation and median (p25 – p75).

⁎ n = 92.

Mean energy intake, considering both FFQ and FR, was 2134.69 kcal/day ±630.16. However, this measure was not included in the statistical analysis as an independent variable to investigate the body fat percentage.

No correlation was found between the continuous variables body fat percentage and mean blood phe levels (*r* = 0.0059).

The univariate analysis of the association between body fat percentage and the categorical variables sex, physical activity level, adequacy of mean blood phe, BMI and stage of sexual maturity showed statistical significance for sex, physical activity level, and BMI. Body fat percentage did not correlate with compliance to treatment (*p* = .706) or pubertal stage (*p* = .515) (Table 3).

**Table 3:** Comparison between body fat percentage and categorical variables sex, physical activity level, adequacy of mean blood phe, BMI, and stage of sexual maturity in adolescents with PKU.

Categorical variable		% body fat	p
Sex^b^	Female	22.90 (19.55–28.75)	< 0.001^a^
	Male	12.20 (8.35–18.90)	
Physical activity level^d^	Active	10.63 ± 4.47	0.114^a^
	Sedentary	18.87 ± 8.86	
Mean blood phe (μmol / L)^c^	Adequate	18.14 ± 9.25	0.706
	Inadequate	18.83 ± 8.48	
BMI^d^	Underweight	11.30 ± 7.57	< 0.001^a^
	Normal weight	16.41 ± 7.65	
	Overweight	26.60 ± 6.06	
	Obesity	30.87 ± 4.64	
Sexual maturity^c^	Prepubertal	20.90 ± 7.37	0.515
	Pubertal	18.46 ± 8.93	

BMI: body mass index. Physical activity level “active” included: “moderately active,” “active,” and “very active.” Data reported as mean ± standard deviation and median (p25 – p75).

a Statistically significant variable in the univariate stage (*p* < .20); included in the multivariate linear regression.

b Mann-Whitney.

c T-Student.

d One-Way ANOVA.

The univariate analysis applied to the continuous variables showed that body fat percentage significantly correlated with BMI (*r* = 0.516, *p* < .001), PAQ-C score (*r* = −0.145, *p* = .167), percentage of total protein intake according to the FFQ (*r* = 0.196, *p* = .058), and mean total protein intake in g/kg/day according to both dietary survey methods (FFQ and FR). The mean total protein intake in g/kg/day from both methods was included in the multivariate stage due to collinearity between FFQ and FR (*r* = 0.797, p < .001).

By the same token, only the categorical variables were included in the multivariate stage even though the categorical and continuous variables for BMI and physical activity level significantly correlated with body fat percentage. The reason was that both types of variables implied similar results.

The following variables were included in the multivariate stage: BMI classification, percentage of total protein intake in the FFQ, mean total protein intake in g/kg/day from both methods (FFQ and FR), PAQ-C score, and sex.

The multivariate linear regression test showed that overweight and obesity, percentage of total protein intake in the FFQ and female sex accounted for 94.1% of the variability in body fat percentage (see Table 4). Obesity was the factor that most strongly correlated with increased adiposity; it was followed in order by overweight, sex and percentage of total protein intake.

**Table 4:** Variables associated with body fat percentage in 10- to 20-year-old individuals with PKU.

	β	95% CI	p	r^2^
Overweight	11.024	7.74–14.30	< 0.001	0.941
Obesity	14.540	10.77–18.30	< 0.001	
Percentage of protein intake (FFQ)	0.169	0.012–0.326	0.036	
Sex (female)	9.703	8.23–11.18	< 0.001	

FFQ: Food Frequency Questionnaire. Multivariate linear regression model. β: regression coefficient; r^2^ determination coefficient.

Complementary analyses showed a moderate positive correlation between body fat percentage and stage of sexual development in the female group (r = 0,402; p = 0,011), while for males this was a negative moderate correlation (r = −0,358; *p* = 0,012). No difference was found for physical activity levels according to sex, but males scored higher than females when comparing the PAQ-C scores (*p* = .008). A moderate negative correlation (*r* = −0.111; *p* = .003) was found between age and physical activity levels, indicating that older adolescents are less active.

## 4 Discussion

Overweight and obesity were the variables most significantly associated with body fat percentage in the sample. Such an association should be expected, as BMI is an important predictor of adiposity and a widely used tool for nutritional diagnosis in clinical practice [24]. Being female contributed to increased fat percentage in adolescents with PKU, and two possible explanations for this finding are biological aspects, namely the female reproductive function and higher estrogen levels [9,25].

In opposition to our findings, some recent data published in the scientific literature have suggested increasing protein intake as a weight loss strategy for healthy adults [26]. Nevertheless, other studies have reported a positive association between protein intake and body fat gain in children because of a stimulus to IGF-1 production and consequent insulin release, which increases total weight [27,28]. To the best of the authors' knowledge, no study has investigated high protein intake in adolescence and its impact on body composition. Unlike adulthood, adolescence is a time of intense growth and high production of anabolic hormones such as GH and IGF-1 [24], which may produce a similar response to that found in children. However, even if the present findings for protein intake in the multivariate analysis provide significant statistical result, this evidence do not support the clinical conclusion that protein intake does have an impact on body fat percentage. A factor that may have influenced these results is the absence of a validated FFQ specific for individuals with PKU. Moreover, some adolescents tend to seek self-affirmation, which may lead to nutritional transgressions and under-reporting of inappropriate food intake [29]. This latter factor seems to be reinforced by the small percentage of adolescents with good phe level control, which evidences the low compliance with dietary recommendations. Another widely discussed aspect is a tendency to under-report food consumption among overweight and obese individuals [30], which may have clouded the results.

Data analysis pointed to a tendency of the FFQ to overestimate food intake, which may account for a lower percentage of protein intake when compared to the FR, a finding already described in the literature [31].

The prevalence of excess weight (overweight and obesity) in individuals with PKU in the present study, 19.14%, was lower than the one found in the population-based study ERICA [32], where 25.5% of Brazilian adolescents were obese or overweight. However, when analyzing obesity separately, there is a similarity between these two samples (8.5% in adolescents with PKU versus 8.4% in the general population of Brazilian adolescents). When it comes to other populations with PKU, the present study also found a lower prevalence of excess weight, once the literature shows a prevalence of 22.0% in Southern Brazil, 15.0% of which are related to overweight and 7.0% to obesity [33].

These findings corroborate a recent systematic review that showed lower rates of overweight compared to the general population while investigating the long-term growth of children and adolescents with PKU [34]. Nevertheless, some studies suggest a higher prevalence of excess weight in patients with PKU [3,35]. An international multicenter study conducted in European countries and Turkey found a rate of combined overweight/obesity among adolescents with PKU of 45.0% in the Netherlands, even though in Turkey the prevalence was only about 19.8%, which is very similar to our findings [36]. A reason for such differences may be related to the strict follow-up carried out by the multidisciplinary team at the UFMG Special Service of Genetics since childhood. As previously demonstrated, higher staffing intensity during treatment is associated with better adherence to PKU medical recommendations [29], hence nutritional recommendations as well. A second possible reason is the refusal of overweight and obese patients to participate in this study because they felt uncomfortable about having their body composition assessed.

Among the studies that used the PAQ-C questionnaire to evaluate physical activity levels in Brazilian adolescents, the present investigation found a higher prevalence of physical inactivity, 96.7%, when compared to 93.5% and 83.0% in the Northeast and Southeast regions of Brazil, respectively [19,37]. Low socioeconomic status negatively influences physical activity [38], and although this aspect was not investigated in this study, part of the individuals with PKU at the UFMG Special Service of Genetics is known to be socioeconomically disadvantaged. Moreover, this finding of a low rate of physical activity may be related to aspects such as stigma surrounding PKU, lower executive function, lower social interaction because of these individuals' restricted diet, and, as a consequence, decreased motivation to engage in physical activities [11,39].

Low dietary compliance was expected to result in higher body fat percentages as indicative of food transgression and high calorie intake, as suggested by some authors [7,8,13]. However, this did not seem to have influenced body fat percentage in the sample, and making a dietary control near the date of the blood exam may have been a contributing factor to this lack of correlation.

The classification of pubertal stage can be seen as a distinguishing feature of this study. The absence of studies that have determined the sexual maturity of their sample may be the reason for the current lack of consensus about overweight among individuals with PKU [33]. This study also stands out for its sample size (*n* = 94), which represents 77% of all adolescents with PKU treated in the state of Minas Gerais. However, some limitations must be taken into account, such as the small sample of prepubertal adolescents, which prevents a deeper, more accurate investigation of the association between sexual maturity and overweight. Furthermore, there was no sample differentiation based on the PKU severity, an important factor that can impact diet profile and treatment compliance.

According to this study, there is no consistent evidence to associate the particularities of the PKU diet and adherence to treatment to the occurrence of overweight and obesity in this population. The levels of physical activity among adolescents with PKU are very similar to those observed in the general population, but the number of sedentary individuals might be higher because of the stigma of the disease in conjunction to socioeconomic deprivation. Thus, it would be advisable for healthcare providers to stress the importance of physical practice during this period of life, based on international recommendations from the WHO. Girls were more likely to have high body fat percentage than boys, and BMI seems to be a good predictor of adiposity in this population, as expected. Better results could be achieved if diet, body composition and physical activity level outside of school could be investigated in a comparative study with a control group.

This research did not receive any specific grant from funding agencies in the public, commercial, or not-for-profit sectors.
